# Outcomes and device use in children with bone-conduction hearing devices in South Africa

**DOI:** 10.4102/sajcd.v71i1.1005

**Published:** 2024-02-19

**Authors:** Chéri van Zyl, Christine Rogers, Silva Kuschke

**Affiliations:** 1 Department of Audiology, Red Cross War Memorial Children’s Hospital, Cape Town, South Africa; 2 Department of Health and Rehabilitation Sciences, Faculty of Health Sciences, University of Cape Town, Cape Town, South Africa; 3 HearX Group, Pretoria, South Africa

**Keywords:** paediatrics, bone conduction hearing devices, outcomes, caregiver feedback, teacher feedback, PEACH, TEACH

## Abstract

**Background:**

Bone-conduction hearing devices (BCHD) can provide hearing solutions in settings where middle ear pathology is rife.

**Objectives:**

Describe functional hearing outcomes and device use of children fitted with BCHD.

**Method:**

Retrospective review of 79 children fitted with BCHD between January 2017 and May 2022. Outcomes included device use and subjective reports measured with the Parents’ Evaluation of Aural/Oral Performance of Children (PEACH) and the Teachers’ Evaluation of Aural/Oral Performance of Children (TEACH). Analysis of variance established association between mean data logging and type and degree of hearing loss. Thematic analyses were done for qualitative outcomes.

**Results:**

Average usage was 7.0 h/day (5.4 SD; range 0.1–24). PEACH ratings indicated 93.3% of children wore their BCHD ‘always’ or ‘often’, with 80% displaying Typical auditory performance at 1-month follow-up. TEACH ratings indicated 84.2% of children wore their BCHD ‘always’ or ‘often’, with 78.9% showing typical auditory behaviour. Increased usage was noted for conductive, mixed, moderate and severe hearing losses. There was a mean delay of 17.2 months (23.4 SD; range 0–90) between age of diagnosis and fitting. Thematic analyses identified two main themes: advantages and barriers to BCDH use.

**Conclusion:**

Average device use fell short of the internationally recommended 10 h/day. Higher BCHD use was associated with higher functional listening performance scores. Long waiting times for medical or surgical intervention for conductive hearing losses can delay BCHD fitting.

**Contribution:**

Limited information is available to examine outcomes in children fitted with BCHD.

## Introduction

The World Health Organization (WHO) has estimated that hearing loss is the most prevalent disabling sensory condition globally, affecting approximately 34 million children (Mahomed-Asmail et al., 2016; WHO, 2016, 2021). Hearing loss is up to four times more prevalent in low-to-middle-income countries (LMICs) compared to more developed countries (Mathers et al., 2000; Swanepoel et al., 2009). Probable factors include the considerable healthcare challenges associated with low-resourced countries, such as underdeveloped healthcare infrastructure, limited availability of hearing healthcare services, pervasive economic hardship, and a heightened prevalence of infectious diseases (Swanepoel & Störbeck, 2008). The WHO approximates that at least 60% of hearing loss in children under the age of 15 years is preventable, with this figure being substantially higher in LMICs (WHO, 2016; 2021). The leading factors contributing to childhood hearing loss in low-resourced areas are associated with otitis media (OM) (57.1%) and congenital abnormalities (21.1%) (Olusanya et al., 2018). Hearing healthcare services in resource-constrained areas are often not prioritised by health systems that are over-burdened by life-threatening diseases (Swanepoel et al., 2010; WHO, 2021). Poor hearing healthcare infrastructure and resources (including a lack of skilled personnel and equipment), as well as geographical barriers, lead to reduced access to hearing healthcare services (Swanepoel et al., 2010; WHO, 2021).

Hearing impairment can have far-reaching consequences on various aspects of childhood development, encompassing language skills, communication abilities, as well as social and emotional well-being (WHO, 2021). Difficulty in effective communication can have detrimental effects on a child’s overall quality of life, resulting in solitude, seclusion, self-consciousness, and frustration (WHO, 2021). In resource-constrained areas, children with hearing loss rarely receive optimal educational opportunities (Mulwafu et al., 2016; WHO, 2021), which directly alters a child’s developmental trajectory. Timeous and targeted hearing intervention can limit the impact of childhood hearing loss (Joint Committee on Infant Hearing [JCIH], 2019).

Children with hearing loss, regardless of degree or type of hearing loss, require auditory support by means of amplification (Bagatto et al., 2011). Research shows that children fitted with amplification at an early age, who undergo rehabilitation, are able to adequately develop speech recognition and language development, particularly when the amplification is fitted appropriately (Ontario Ministry of Children and Youth Services, 2019). Various guidelines, such as the *American Academy of Audiology Paediatric Amplification Protocol* (American Academy of Audiology, 2013) and the National Institute for Health and Care Excellence (NICE) *Guideline concerning Otitis Media with effusion in children under 12 years of age* (NICE, 2008), endorse the use of appropriate hearing technology in conjunction with evidence-based fitting procedures for hearing devices (American Academy of Audiology, 2013). Children who receive a diagnosis of permanent hearing loss, starting from a mild degree, should, whenever anatomically feasible, be provided with air conduction hearing aids (American Academy of Audiology, 2013). However, in cases where this is not possible (i.e., atresia, chronically discharging ears, additional structural malformations), bone conduction hearing devices (BCHDs) are recommended (American Academy of Audiology, 2013).

Bone conduction hearing devices serve as alternative amplification choices for children facing both transient and enduring conductive hearing loss (CHL). These devices facilitate sound transmission directly to the cochlea by sending vibrations from a sound processor to the mastoid, effectively circumventing the external or middle ear (Westerkull, 2018). Bone-conduction hearing devices are beneficial in CHL as they provide continuous, quality access to sound even when air conduction thresholds fluctuate (Westerkull, 2019). In recent years, BCHDs have also become a common treatment option for children with unilateral severe to profound sensorineural hearing loss (SNHL) (Dornhoffer & Dornhoffer, 2016). The BCHD transmits the auditory stimulus via the skull to the inner ear and cochlea of the other, normal hearing ear (Doshi et al., 2016; Reinfeldt et al., 2015).

There is a need for outcomes-based intervention in the paediatric population to provide clinicians with insight on whether a child is benefiting from their hearing technology and by how much (Cox et al., 2000; Gatehouse, 2003). Several approaches are available when evaluating the efficacy of hearing technology in the paediatric population, and can be grouped into objective and subjective measures (Cox et al., 2000; Kramer et al., 2002). Objective measures are usually carried out in a controlled environment with a focus on speech recognition in various clinically created scenarios. However, there is concern that this form of measurement may not provide realistic information for experiences of daily listening situations (Haggard et al., 1981; Oja & Schow, 1984). To try and avoid these limitations, subjective measures can be used. However, in the paediatric population, obtaining true subjective information is limited, and informal observations are difficult to interpret with regard to direct hearing benefit (American Academy of Audiology, 2013). Hence, measures through self-assessment inventories such as the Parents’ Evaluation of Aural/Oral Performance of Children (PEACH) and Teachers’ Evaluation of Aural/Oral Performance of Children (TEACH) are used as they attempt to quantify functional benefit in daily listening conditions from those closest to the child. Subjective measures have gained traction in the last few years as they can be completed easily within a session, and capture real life data (Bagatto et al., 2011; Stelmachowicz, 1999).

There is a lack of consensus on what constitutes typical outcomes for infants or children who wear hearing technology, or how a hearing health professional should monitor auditory development and achievement over time (Bagatto et al., 2011; Saunders et al., 2005). The method of determining amplification efficacy in young children is multifaceted. Continued research into paediatric hearing loss and outcome measures in audiological practice is essential to profession growth and person-centred care. Recently, three studies in South Africa looked at the outcomes of children with hearing loss; however, all focused on children fitted with behind-the-ear hearing aids (Booysen et al., 2021; Kuschke et al., 2021; Van Zyl et al., 2022). In low-resourced contexts, where middle ear pathologies and various syndromes associated with hearing loss are common (Kesser et al., 2013), data on the context-specific functional hearing outcomes for children who wear BCHD is necessary. This study describes the functional hearing outcomes of children fitted with BCHDs on soft-bands through their average daily device use, as well as caregiver and teacher feedback on auditory performance.

## Methods

### Setting

Red Cross War Memorial Children’s Hospital (RCWMCH) is the first stand-alone tertiary hospital in sub-Saharan Africa dedicated entirely to child health care. It serves as a central referral hospital for paediatric patients across the entire Western Cape who require specialised healthcare services. The hospital predominantly serves children from the public health sector. The RCWMCH Audiology Department provides hearing healthcare services for approximately 300 children on a monthly basis.

### Study design and population

A retrospective examination of clinical records encompassing children aged 0–13 years who received BCHD devices from January 2017 to May 2022 was undertaken. Children were included in the study if they met the criteria of receiving a BCHD on a soft-band and had data-logging records at the 1-month follow-up. Additionally, any qualitative information gathered from parent and teacher questionnaires at the 1-month follow-up was also documented.

### Data collection materials and procedures

Participants were identified retrospectively using an electronic departmental database from which their demographic information (including age of suspicion and diagnosis of hearing loss, age at BCHD fitting, type and degree of hearing loss) was recorded. Clinical records were reviewed to obtain basic demographic information, BCHD information, and information recorded on validation questionnaires.

The average daily device usage (h/day) was documented by capturing the data-logging information recorded at the 1-month follow-up appointment. All patients were fitted unilaterally regardless of bilateral hearing loss (due to resource limitations), and data-logging was recorded for the ear fitted. Additionally, clinical records were reviewed for each participant who attended their 1-month follow-up to obtain their BCHD validation information measured either through the PEACH or TEACH questionnaires.

The PEACH and TEACH assessments gauge everyday functional auditory and communication performance in children (Cupples et al., 2018; Marnane & Ching, 2015). These tools can help pinpoint situations that might disrupt a child’s consistent use of amplification devices (Marnane & Ching, 2015). Caregivers or teachers are tasked with observing and evaluating a child’s listening and communication abilities in both quiet and noisy real-life scenarios (Ching & Hill, 2007; Ching et al., 2018). Moreover, they were designed to cater to children of all age groups (Ching & Hill, 2007).

Responses to these questionnaires are categorised using a five-point scale: 0 for ‘Never or 0% of the time’, 1 for ‘Seldom or 25% of the time’, 2 for ‘Sometimes or 50% of the time’, 3 for ‘Often or 75% of the time’, and 4 for ‘Always or greater than 75% of the time’ (Ching & Hill, 2007). The PEACH questionnaire comprises 13 items, while the TEACH questionnaire has 11 items. A performance score is then calculated for quiet, noisy, and overall environments by summing the values of all items. These scores are expressed as percentages, with a higher percentage indicating more favourable listening outcomes (Ching & Hill, 2007; Wong et al., 2018). The cumulative percentage scores for each subcategory can be used to determine auditory behaviour as ‘typical performance’, ‘possible review indicated’, or ‘further review indicated’ (Bagatto et al., 2011). This evaluation can be conducted by audiologists or other healthcare professionals (Ching et al., 2018).

The questionnaires were used in their original English format and were issued to caregivers in hard copy at the BCHD fitting. Caregivers and teachers were encouraged to observe the child’s behaviour during the first month with amplification and complete the questionnaires in the week before their appointment. The managing audiologist scored and recorded the returned questionnaire at the 1-month post-fitting appointment. If caregivers were not proficient in English, the managing audiologist administered the PEACH questionnaire in an interview format. There is a section for additional comments at the end of both questionnaires, allowing for qualitative written feedback to be obtained by caregivers and teachers. These were recorded for thematic analyses.

### Data analysis

All data were captured on a Microsoft Excel spreadsheet, (Microsoft Corp 2022, Redmond, WA). The data were analysed using Statistical Package for Social Sciences (SPSS) version 27.0 (IBM Corps., Armonk, NY). Descriptive (percentages, measures of central tendency and measures of variability), as well as inferential statistical methods were used to analyse quantitative data. The students t-test (*p* < 0.05) was used to compare average device use between typically performing children and those who required review based on the PEACH and TEACH questionnaire scores. Analysis of variance (ANOVA) was used to compare mean data logging to the type and degree of hearing loss. For both the PEACH and TEACH questionnaires, percentage scores for the three domains were calculated according to the score key. Top-down, deductive thematic analysis was conducted on the qualitative information obtained from the written feedback section on both the questionnaires (Braun & Clarke, 2006). The researchers manually inputted qualitative written data into an Excel spreadsheet, whereafter the data were categorised, coded, and grouped according to identified themes by Researcher 1, and checked and moderated by Researchers 2 and 3.

### Ethical considerations

Ethical clearance to conduct this study was obtained from the University of Cape Town Faculty of Health Sciences Human Research Ethics Committee. (No. HUM217/2022) and the Red Cross War Memorial Children’s Hospital (RCWMCH) Research Review Committee (No. RCC329 / WC_202204_034).

## Results

A total of 500 children were fitted with amplification devices between January 2017 and May 2022 at RCWMCH. During this period, 116 children were fitted with a BCHD, of which 79 children were included in the study sample. In all, 37 children were excluded from this study sample as they had no documented BCHD information, and no information was recorded on the validation questionnaires. The study population characteristics are presented in Table 1. Most participants (73.3%) attended a mainstream school, and nearly 70% came from a low-income household (*n* = 79). Almost half of the sample (45.6%) had normal hearing unilaterally. The most common aetiological factors associated with hearing loss were ENT-related pathology (49.4%) and syndromic causes (26.5%).

**TABLE 1:** Characteristics of study population (*N* = 79).

Variable	%	*n*
**Gender**		
Male	41.8	33
Female	58.2	46
**Age (school phase)**		
0–4 years (before school)	17.1	14
5–8 years (foundation phase)	32.9	26
9–11 years (intermediate phase)	40.5	32
12–13 years (senior phase)	8.9	7
**Household income**		
H0 (formally unemployed)	19.0	15
H1 (0 USD–489.31 USD per month)†	69.6	55
H2 (489.31 USD–1712.62 USD per month)†	7.6	6
H3 (>1712.62 USD per month)†	3.8	3
**HIV-status**		
HIV-positive	10.1	8
HIV-negative	89.9	71
**Home language**		
Afrikaans	27.8	22
English	38.0	30
Xhosa	29.1	23
Other	5.1	4
**Educational setting**		
Too young for formal schooling	7.9	10
Special education needs school	5.0	4
Hearing-impaired skills school	3.8	3
Hearing-impaired mainstream school	5.0	4
Mainstream school	73.3	58
**Aetiological factors associated with hearing loss**		
Neurological	1.3	1
Infectious	2.6	2
Syndromic	26.5	21
Trauma	3.8	3
ENT	49.4	39
None	16.4	13

HIV, human immunodeficiency virus.

† , Exchange rate of 1 USD = R17.03 (South African Rand/ZAR).

**FIGURE 1:**
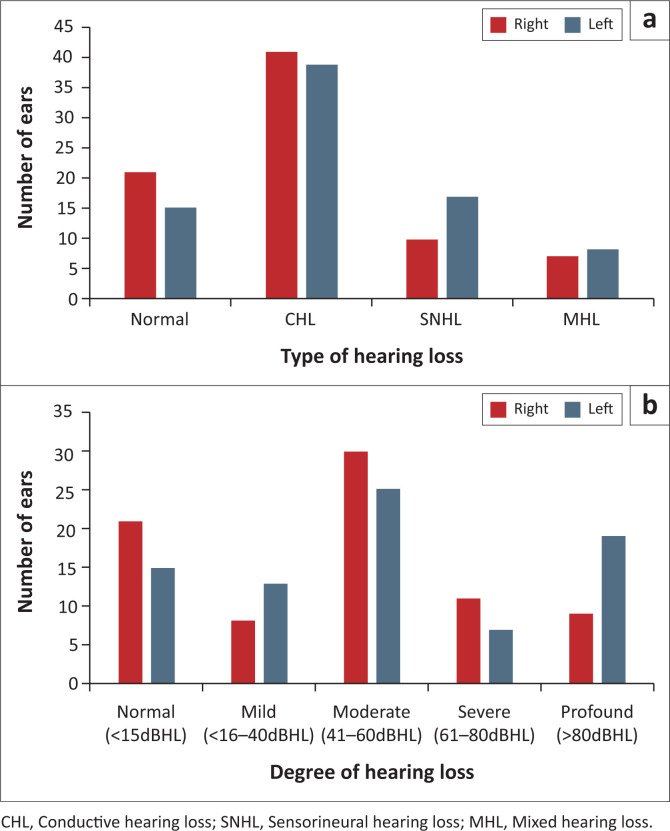
Type (a) and degree (b) of hearing loss per ear (*N* = 158 ears).

Figure 1 shows that more than half (50.6%) of the ears of children in this sample had CHL, with the majority at a moderate hearing level (34.8%).

Table 2 depicts the age of suspicion, diagnosis, and hearing aid fitting for the study population. The mean age at diagnosis was 75.7 months (37.2 SD; range 1–151), with an almost 18-month delay between diagnosis of hearing loss and fitting (17.2 months, 23.4 SD; range 0–90).

**TABLE 2:** Age of suspicion, diagnosis, and hearing aid fitting (*n* = 79).

Variable	Age of suspicion (months)	Age at diagnosis (months)	Age at fitting (months)	Diagnosis to fitting delay
Mean; SD	64.5; 42.2	75.7; 37.2	92.9; 38.7	17.2; 23.4
Range	1–151	1–151	6–151	0–90

SD, standard deviation.

### Bone-conduction hearing device use

Table 3 illustrates the mean data logging for the overall group (*n* = 79) which was 7.0 h/day (5.4 SD; range 0.1–24) at the 1-month follow-up appointment. Based on PEACH and TEACH performance indicators, no statistically significant differences were found between Typical Performance and Review Groups (*p* = 0.188).

**TABLE 3:** Data logging for typical performance and review groups as per Parents’ Evaluation of Aural/Oral Performance of Children and Teachers’ Evaluation of Aural/Oral Performance of Children.

Variable	Mean data logging in hours/day	SD	Range	*p*
Overall (*n* = 79)	7.0	5.4	0.1–24	-
Typical performance (*n* = 27)	7.4	3.8	0.6–12.3	0.188
Review required (*n* = 7)	4.9	4.1	0.3–12.2	-

SD, standard deviation.

### Relationship between bone-conduction hearing device use and hearing loss type and degree

**FIGURE 2:**
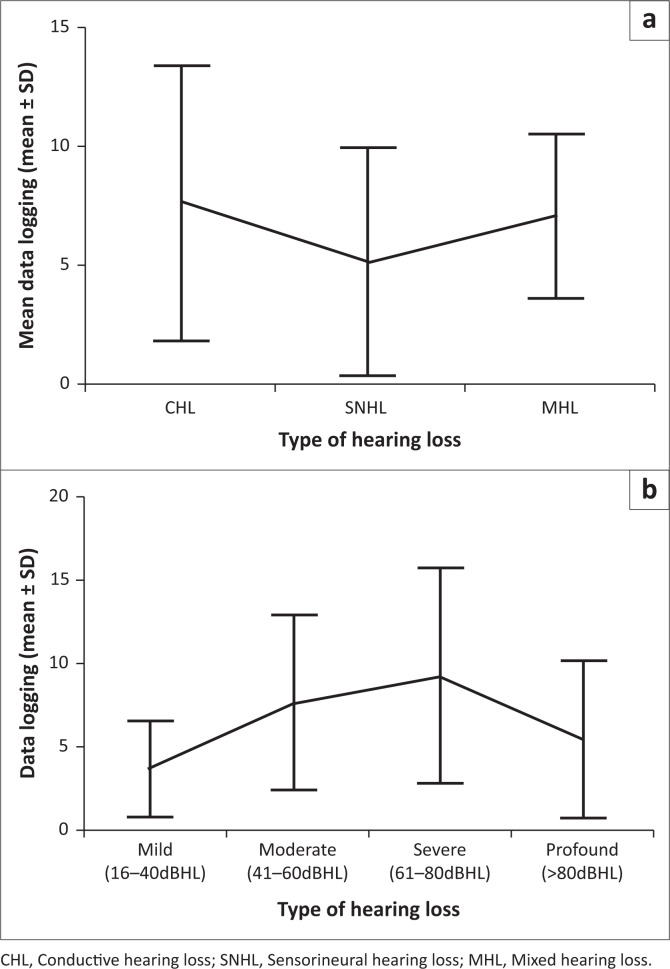
Relationship between data logging and hearing loss type (a) and degree (b) (*n* = 79).

Figure 2 shows that participants with CHL had the highest mean data logging (7.7 h/day, 5.8 SD; range 0.2–24.0), whereas the lowest mean data logging was found in participants with SNHL (5.2 h/day, 4.8 SD; range 0.0–16.9).

The highest mean data logging was recorded for participants with severe hearing loss (9.3 h/day, 6.5 SD; range 0.6–21.5). Mean data logging increased along with the severity of hearing loss, except in those with profound hearing loss, where mean data logging decreased to 5.5 h/day (4.7 SD; range 0.0–16.9).

No statistically significant differences were found between mean data logging and type or degree of hearing loss (*p* = 0.193 and *p* = 0.051, respectively).

### Outcomes reported by caregivers and teachers

Caregivers and teachers observed the children’s behaviour in the month after the initial device fitting. They completed the PEACH and TEACH questionnaires in the week before their 1-month follow-up appointment. The PEACH and the TEACH questionnaires were brought back to the 1-month post-fitting follow-up appointment by caregivers for 15 and 19 participants respectively.

**FIGURE 3:**
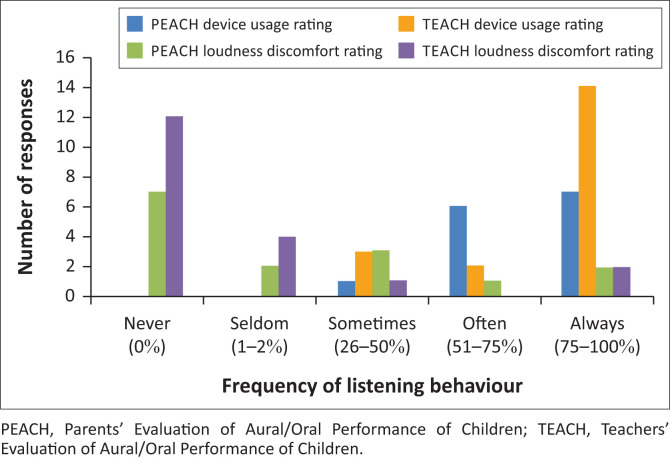
Caregiver (*n* = 15) and teacher (*n* = 19) reported ratings for children’s bone-conduction hearing device usage and loudness discomfort levels.

**FIGURE 4:**
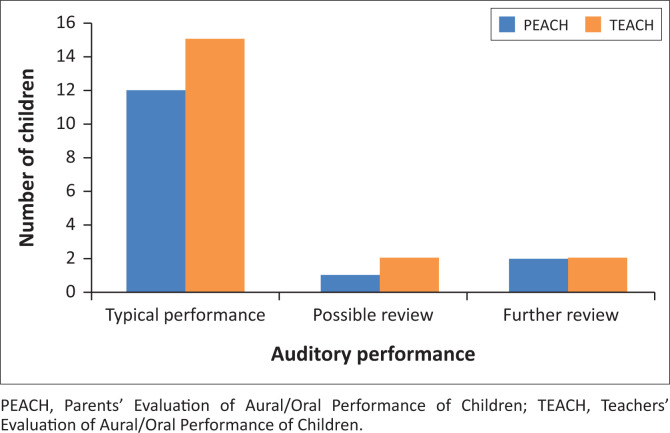
Performance indications for children using bone-conduction hearing devices as rated by caregivers and teachers (*n* = 34).

As illustrated in Figure 3 and Figure 4, PEACH ratings indicated that 93.3% of children wore their BCHD always or often, with 80.0% displaying typical auditory performance 1-month post-fitting. Teachers’ Evaluation of Aural/Oral Performance of Children ratings indicated that 84.2% of children wore their BCHD always or often at school, with 78.9% displaying typical auditory behaviour.

Both caregivers and teachers indicated that the children performed better in quiet scenarios than in noise (Table 4).

**TABLE 4:** Mean Parents’ Evaluation of Aural/Oral Performance of Children and Teachers’ Evaluation of Aural/Oral Performance of Children percentage (%) scores for quiet, noise, and overall environments.

Variable	Mean	SD	Range
**PEACH (*n* = 15)**			
Quiet	84.2	14.6	50.0–100.0
Noise	79.9	18.7	45.0–100.0
Overall	81.8	15.2	50.0–100.0
**TEACH (*n* = 19)**			
Quiet	87.6	11.8	60.0–100.0
Noise	77.2	16.8	43.8–100.0
Overall	82.8	13.1	58.3–100.0

PEACH, Parents’ Evaluation of Aural/Oral Performance of Children; TEACH, Teachers’ Evaluation of Aural/Oral Performance of Children; SD, standard deviation.

Caregiver (*n* = 9) and teacher (*n* = 12) written feedback from the *additional comments* section of the PEACH and TEACH questionnaires was obtained. All comments were included in the thematic analysis, and two themes were extracted: advantages and barriers to BCHD usage. There were seven sub-themes identified, three under advantages and four under barriers. These are summarised with examples in Table 5.

**TABLE 5:** Thematic analysis of caregiver and teacher feedback regarding bone-conduction hearing devices usage (*n* = 20).

Advantages	Examples and/or illustrative quotes
**Sub-theme**	
Improved listening	‘Enjoys TV and listening to stories being told’.
	‘Not complaining anymore’.
	‘His hearing/listening behaviour is much better now ever since wearing his hearing aid. He can now answer you quick something that he were used to struggle on before he got his hearing aid. Even in a noisy place he does hear quick and respond quickly’.
	‘No longer complains about how she cannot hear me or what others are saying. She responds to everything she hears, even to soft sounds’.
	‘She most times executes her activities without asking me to repeat it’
	‘Made a world of difference on how he could hear’
	‘The child is hearing properly’
	‘He responds when talking to him and follow instructions’
	‘The learner responds to instruction given in a quiet environment’
Improved behaviour	‘He can sit and work quietly in a noisy environment’
	‘Behaviour at home changed, to being more calm and sociable with family and friends. He is more eager to do his homework’
	‘Talks a bit more and is more out of her shell’
	‘She is participating well in class. She behaves well’.
	‘The BCHD are really helping him a lot in class and active in his schoolwork. He has become a more confident boy’.
	‘She use to be very quiet in class, but since she have this hearing band her work in class has improved very well’
Acceptance	‘His siblings has also been amazing making him feel comfortable’
	‘I encouraged him to wear it… Secondly other learners did not respond the way he anticipated and that encourage him to wear his aid all the time’
**Barriers**	
**Sub-theme**	
Compliance	‘At home she forget to wear a hearing aid’
	‘He does often suffer from headaches’†
Bullying	‘Sometimes it’s hard when he plays outside with other kids, when they touch it he tells them don’t take my belt I need it more than you do’
	‘Initially he was uncomfortable wearing his hearing aid feeling/thinking that other learners would make fun of him’
Sound quality	‘Complaining about the voices. He is complaining about sound’
	‘She does however, find it very difficult to follow instructions given in a noisy environment and relies on hand signals and looking at my mouth for confirmation of the instructions’
Query need for BCHD	‘Hearing aid irritates her because she is always trying to pull it off’
	‘I have not noticed any difference in his behaviour or ability to hear’

BCHD, bone-conduction hearing devices.

† , The authors have deduced that the patient has headaches when wearing the soft-band, hence inclusion here as a barrier to compliance.

## Discussion

This study aimed to describe the functional hearing outcomes and device use in a cohort of children fitted with BCHDs between January 2017 and May 2022 at a tertiary level public hospital in South Africa.

Access to quality sound is crucial for the development of spoken language, academic performance, and socio-emotional well-being (Yong et al., 2020). In particular, children with persistent CHL have limited vocabulary, poor attention to auditory stimulation, and face difficulty with speech perception and reading (Rosenfeld et al., 2016; Smit et al., 2021). Previous studies indicated that young children with unilateral atresia had no grade failure compared to their typical peers if they consistently used hearing technology (Kesser et al., 2013; Smit et al., 2021). It has been recommended that children with Down Syndrome who have a genetic predisposition for CHL be fitted with hearing aids as part of the standard of care (Austeng et al., 2013).

Hearing technology is only effective if consistently used (Gan et al., 2017). Average data logging was 7.0 h/day for the sample in this study, which is higher than reported device use with air-conduction hearing aids in three previous South African studies (Booysen et al., 2021; Kuschke et al., 2021; Van Zyl et al., 2022), but still falls short of the daily recommended average device use of 10 h/day for optimal speech- and language development through audition (Tomblin et al., 2015).

There was a mean delay of 17.2 months between the age of diagnosis and the age of BCHD fitting in this sample. Possible reasons for the mean delay of almost a year and a half could pertain to first-line medical or surgical treatments for middle ear pathology before amplification is considered, as well as long waiting lists for specialised ENT consultations at tertiary level hospitals. While some of the data collection period fell within the coronavirus disease 2019 (COVID-19) pandemic, the audiology department was only closed for the initial ‘hard lockdown’ in March 2020; thereafter services resumed. Therefore, it is unlikely that this had any impact on the delay between age of diagnosis and age of fitting of the BCHD. The NICE (2008) recommends fitting hearing aids for children with OM with effusion that does not resolve in 3 months or as an alternative to grommet insertion. Additionally, hearing aids are recommended for children awaiting surgery for middle ear pathologies to mitigate the adverse effects of temporary hearing loss on speech and language development and academic performance (National Institute for Health & Care Excellence, 2008).

Device use was higher for children in the *typical* functional listening performance group (7.4 h/day) than for the group who required review (4.9 h/day) based on their PEACH and TEACH scores. Although device use was clinically higher for the *typical* performance group, there was no statistically significant difference in device use between the two groups. In a previous study on hearing aid and cochlear implant usage in young children, higher PEACH scores were associated with higher device use (Marnane & Ching, 2015). Possible reasons for the lack of statistical significance in the current study were small group sizes and the number of patients per group that were not equally distributed.

The primary aetiology associated with hearing loss was middle ear pathology (49.4%) and more than a quarter of the children in this sample were syndromic (26.6%). More than half of the ears in this sample (50.6%) presented with CHL at a moderate (34.8%) hearing level. The children in this sample were reflective of the low-resourced context, where middle ear pathology and syndromes occur more commonly (Olusanya et al., 2018). Patients with CHL wore their BCHD for more average hours (7.7 h/day) than patients with SNHL (5.2 h/day). Mean data logging increased as the severity of hearing loss increased, except in patients with profound hearing loss, where device use decreased. Bone-conduction hearing devices bypass the middle ear and are typically indicated for use in patients with conductive and mixed hearing losses (Liu et al., 2017).

Most caregivers (93.3%) for the PEACH group (*n* = 15) and most teachers (84.2%) for the TEACH group (*n* = 19) reported that children wore their BCHD always or often; however, the average data logging hours for the overall group was only 7.0 h/day. Parents and teachers frequently over-estimate subjective device use compared to data logging information documented within the hearing device (Walker et al., 2015).

Both parents and teachers reported *typical* functional auditory performance in nearly 80% of the sample. Possible reasons for the high *typical* performance scores include the high number of unilateral normal hearing ears (45.6%), and the fact that most participants (73.3%) attended mainstream schools. Both parents and teachers reported higher questionnaire scores in quiet scenarios than in noise. Higher PEACH scores in quiet situations were also reported in a previous South African study involving young children using air-conduction hearing aids (Kuschke et al., 2021). Noisy environments at home and in educational settings are detrimental to listening and learning opportunities for children with hearing loss, regardless of the type or degree of hearing loss (Benítez-Barrera et al., 2020). Strategies such as the use of FM systems should be implemented in educational settings to reduce the adverse effects of noisy classrooms (Benítez-Barrera et al., 2020).

Qualitative caregiver and teacher reported feedback included advantages of BCHD use, such as improved listening and behaviour at home and school. Caregivers and teachers noted positive changes in children’s confidence, attention, and social engagement within 1 month of BCHD fitting. The positive caregiver and teacher reports allude to visible advantages that can be reported only a few weeks post-fitting. Reported barriers to BCHD use included compliance, issues with sound quality. and bullying. Hearing healthcare professionals play a pivotal, collaborative role with parents and teachers to address ongoing hearing device management challenges (Muñoz et al., 2015) in order to facilitate consistent hearing aid use. Sound quality with BCHD could be improved through permanent implantation, negating the transmission of sound through the soft-band, and transmitting the auditory stimulus via the skull directly to the inner ear (Doshi et al., 2013; Reinfeldt et al., 2015). Older children should be empowered to become advocates in their own management plan, which could address factors that influence device use negatively, like bullying (Ida Institute, n.d.).

The wide age distribution in this sample impacted on generalising findings. Future studies on functional outcomes with BCHD use in low-resourced contexts should consider specific age groups, for example, toddlers, preschoolers, and school-aged children. The utilisation of BCHDs and the associated outcomes were documented at a single point in time during the 1-month post-fitting follow-up appointment. Gathering longitudinal data on BCHD usage in resource-limited settings in the future could provide insights into whether device usage increases over an extended timeframe. This longer timeframe could allow for a more comprehensive description of functional outcomes, benefits, and challenges associated with BCHD use.

## Conclusion

Functional listening outcomes of children fitted with BCHD at a paediatric public hospital in South Africa showed average daily device use of 7.0 h/day, which fell short of the 10 h/day international recommendation. Typical aural/oral performance was documented for nearly 80% of the children in this sample, and clinically higher BCHD use was associated with higher functional listening performance scores. Increased daily BCHD use was positively associated with conductive, mixed, moderate, and severe hearing losses. Long delays between hearing loss diagnosis and BCHD fitting could pertain to long waiting times for medical or surgical interventions for conductive and mixed hearing losses. Future longitudinal research on BCHDs should be considered to describe functional outcomes and device use more comprehensively.
